# Decreased expression of miR-195 mediated by hypermethylation promotes osteosarcoma

**DOI:** 10.1515/med-2022-0441

**Published:** 2022-03-07

**Authors:** Tianhao Sun, Dongning Liu, Jun Wu, William W. Lu, Xiaoli Zhao, Tak Man Wong, Zhi-Li Liu

**Affiliations:** Shenzhen Key Laboratory for Innovative Technology in Orthopaedic Trauma, Guangdong Engineering Technology Research Center for Orthopaedic Trauma Repair, Department of Orthopaedics and Traumatology, The University of Hong Kong-Shenzhen Hospital, Shenzhen 518053, China; Research Center for Human Tissue and Organs Degeneration, Institute Biomedicine and Biotechnology, Shenzhen Institutes of Advanced Technology, Chinese Academy of Sciences, Shenzhen 518055, China; Shenzhen Key Laboratory for Innovative Technology in Ortho-paedic Trauma, Guangdong Engineering Technology Research Center for Orthopaedic Trauma Repair, Department of Orthopaedics and Traumatology, The University of Hong Kong-Shenzhen Hospital, Shenzhen 518053, China; Institute of Spine and Spinal Cord, Department of Orthopedic Surgery, The First Affiliated Hospital of Nanchang University, Nanchang 330006, China; Department of Spinal Surgery, Shenzhen Sixth People’s Hospital(Nanshan Hospital), Huazhong University of Science and Technology Union Shenzhen Hospital, Shenzhen, China

**Keywords:** osteosarcoma, fatty acid synthase, miR-195, DNA methylation

## Abstract

Osteosarcoma (OS) is the most common type of primary malignant bone tumor. The early lung metastasis of osteosarcoma is one of the main factors of poor prognosis. Therefore, searching for new targets and new mechanisms of osteosarcoma metastasis is essential for the prevention and treatment of osteosarcoma. Our previous studies suggested that fatty acid synthase (FASN) was an oncogene and promoted osteosarcoma. In addition, it is reported that the expression of miR-195 was negatively correlated with osteosarcoma. Aberrant DNA methylation can reversely regulate the expression of miRNAs. However, whether miR-195 could target FASN in osteosarcoma and whether ectopic DNA methylation is the upstream regulatory mechanism of miR-195 in metastasis of osteosarcoma are not fully studied. The expressions were detected by qPCR and western blot, and methylation level was determined by methylation-specific PCR. Luciferase reporter assay, MTT, wound healing, and Transwell assay were used. We found that the expression of miR-195 was low in osteosarcoma. The methylation of miR-195 was high. miR-195 targeted and decreased the expression of FASN. In osteosarcoma, miR-195 inhibited cell proliferation, cell migration, and invasion. The methylation of miR-195 was related to decreased miR-195, it might promote osteosarcoma.

## 1 Introduction

Osteosarcoma (OS) is the most common bone tumor and the eighth form of cancer in children [1]. It is classified as osteoblastic, chondroblastic, or fibroblastic osteosarcoma [2]. The morbidity of osteosarcoma accounts for 35% of all the primary bone cancers. Recurrence and lung metastasis are the leading cause of death in patients [3]. Migration is the primary step in the process of metastasis, with the main event involving epithelial–mesenchymal transition that allows the cells to become more motile [4]. Metastasis remains the most vital complication of osteosarcoma. The survival rate of patients with metastasis is low [5]. Osteosarcoma usually exhibits locally invasive growth. Pulmonary metastasis is frequently found in patients [6]. While only 20% of patients with metastasis of osteosarcoma are detectable clinically, the majority of the remaining 80% are presumed to have undetectable micro-metastases of lungs at diagnosis [7]. Precision medicine suggests one to find molecules such as proteins or miRNAs as the therapy targets. Thus, searching for effective and sensitive molecular biomarkers for osteosarcoma is urgently needed.

Fatty acids include substantial functions in cells from membrane structure and energy storage to signaling transduction and protein acylation [8]. Fatty acids are the main ingredient of phospholipids and glycolipids, which are also the main ingredient of cell membranes. Glycolipid plays important role in cell growth, cell differentiation, and signal transduction [9]. In human beings, the most common type of fatty acid is palmitic acid. The fatty acid is oxygenated and degraded into acetyl-coenzyme in the mitochondria, which provide energy. A fatty acid is also responsible for the formation of certain second messengers involved in the response to extracellular signals [9].

The fatty acid synthesis pathway has two main functions: one is the storage of excess energy intake such as fat, and the other is the synthesis of fat from carbohydrates or proteins if the diet is low in fat [8]. There are correlations between abnormal fatty acid metabolism and tumor such as unlimited cell proliferation and invasiveness, consistent with the hypothesis that fatty acid metabolism disorder might be one of the factors inducing carcinogenesis [10]. Fatty acid synthase (FASN) is the main synthetic enzyme that produces the free fatty acid palmitate. Because humans consuming a proper diet undergo little endogenous fatty acid synthesis, dietary lipids would provide the necessary fatty acid for survival, growth, and development.

microRNAs (miRNAs) are small, single-stranded RNA regulating gene expression [11]. They regulate substantial physiological and pathological processes including cell proliferation, cell differentiation, and cell death. One miRNA can bind to numerous mRNAs, resulting in the regulation of genes [12]. Thus, aberrant miRNA expression is associated with diseases such as osteosarcoma [13], osteoporosis [14], and osteoarthritis [15]. One of the facts of miRNAs is that they can bind to their target mRNAs and then degrade targets. During the process of degradation, argonaute family proteins play vital roles. Translational repression and 5′-to-3′ mRNA degradation lead to the repression of the target gene expression. Overexpressed miRNAs in malignant cells are related to carcinogenesis, and target and decrease the expression levels of tumor suppressors. In contrast, miRNAs lost by tumors lead to the expression of the oncogene. The next-generation sequencing technologies have made an investigation of miRNA expression profiling easy.

The CpG sites are regions of DNA where a cytosine is followed by a guanine. Cytosine in CpG can be methylated, which can change gene expression. CpG islands also occur frequently in promoters for miRNAs [16,17,18], which inhibit mRNA transcription or induce mRNA degradation and shed new light on cancer research [19,20,21,22,23,24,25]. In cancer, the CpG islands are commonly methylated and the related genes or miRNAs are downregulated.

Reports found that the expression level of miR-195 was negatively correlated with osteosarcoma metastasis, and upregulating miR-195 could inhibit the apoptosis of osteosarcoma cells. Our previous study showed silencing FASN could inhibit cell proliferation and migration of osteosarcoma [13]. However, whether miR-195 could target FASN in osteosarcoma was not fully studied. In this study, we will investigate the relationships between miR-195 and FASN, and the methylation of the promotor region of miR-195.

## 2 Materials and methods

### 2.1 Clinical tissues and cell culture

We selected six fresh osteosarcoma tissues and six normal tissues from clinical tissue biopsy in The First Affiliated Hospital of Nanchang University from September 2020 to July 2021. All patients were selected based on X-ray, CT, bone scan of the body, and pathology methods. Before the collection of samples, the patients were not treated by drugs or radiotherapy. All of the studies were approved by the Ethics Committee of the above hospital (2020) (12–103), and all patients signed the Informed Consent Form. The study methodologies conformed to the standards set by the Declaration of Helsinki. One of our authors had access to information that could identify individual participants during or after data collection. Osteosarcoma cell lines 143B and U2-OS were cultured in DMEM and DMEM/F12, respectively, with 10% FBS and penicillin/streptomycin.

### 2.2 Quantitative real-time PCR (qPCR)

All 100 mg tissues were mixed with 1 mL of TRIzol. The same amount of RNA was used for all the samples. Each cDNA sample was triplicated in 96-well plates. Data were analyzed using the 2^−ΔΔ*C*T^ relative quantification method. U6 was the internal control. The sequences of primers were as follows: miR-195: 5′-UAGCAGCACAGAA AUAUUGGC-3′; U6 forward: 5′-ATGACACGCAAATTCGTGAAGC-3′; miRNAs reverse: 5′-GCGAGCACAGAATTAATACGAC-3′.

### 2.3 Western blot

The lysis buffer was a RIPA buffer with a protease inhibitor cocktail. The cells were added to lysis buffer and subjected to alternating vortexing and then stored at 4°C for half an hour. Then, the cell lysis was performed by centrifugation at 16,000*g* for 20 min at 4°C. Laemmli buffer was used to denature the samples. The PVDF membrane was blocked by 5% BSA solution for 1 h. Primary (first) antibodies included anti-FASN antibody (sc-55580, mouse anti-human, 1:100) and GAPDH antibody (Abcam, ab9485, 1:2,500). The membrane was incubated with the primary antibody overnight at 4°C, rinsed with TBST, and then incubated with the secondary antibody for 1 h at room temperature. ECL Prime Western Blotting Reagents (GE Healthcare) were used to develop.

### 2.4 DNA extraction and methylation-specific PCR (MSP)

A DNA extraction kit (Biomed, Beijing, China) was used to extract DNA from osteosarcoma cells, and the EZ Methylation kit (Zymo Research, Orange, CA) was used for bisulfite modification of genomic DNA (500 ng). The DNA methylation status was analyzed by MSP using primers specific for either the methylated or bisulfate modified unmethylated DNA. Ten microliters of PCR products was loaded onto 6% polyacrylamide gels, electrophoresed, stained with ethidium bromide, and visualized under ultraviolet light. The MSP primers for miR-195 were as follows: left M primer: AGTGTTTTTTGTTTTTAGGAGAGAC, right M primer: TCTAATCTCAACCTTTAATTTCCGT; left U primer: GTTTTTTGTTTTTAGGAGAGATGT; right U primer: TCTCTAATCTCAACCTTTAATTTCCAT.

### 2.5 Luciferase reporter assays

First, we designed sequences including the two sequences of FASN as follows: UTR primers (5′-3′) U: CCCCTCGAGCCTGCCACCGGAGGTCACT, L: CGGGCGGoCGCGTGGGAGGCTGAGAGCAGCA; UTR mutant primers (5′-3′): U: TTATACAAAACAAAAGCGATAA, L: AAGCAACAGAAACCCCCTGT. We inserted the wild type and mutant fragments of the 3′-UTR of FASN containing the binding sites of miR-195 into the luciferase vector. We co-transfected the cells with the above plasmids and miR-195. The transfection method for luciferase assays is according to the instructions of LipofectamineTM 2000 (Lipo2000). One day before transfection, the OS cells in the serum-free medium were seeded in the 48-well plate. For each transfection sample, the following amounts of reagents were used. A total of 100 ng of plasmids psi-FASN/psi-FASNm (V1 = 0.25 µL) was diluted in 25 µL of serum-free medium. At the same time, miR-195 or negative controls (V2 = 6 µL) was diluted in 25 µL of serum-free medium. Lipo2000 (V3 = 1 µL) was diluted in 25 µL of serum-free medium. The above V1, V2, and V3 were mixed and incubated for 20 min, and then added into the cell medium. After 5 h, the above medium was changed to the culture medium. The cells were cultured for 48 h.

### 2.6 Lentiviruses silencing or overexpressing miR-195

For lentiviruses overexpressing miR-195, the vector was pSico-GFP. We first designed sequences *xho*I-U 5′-3′: AATCTCGAGATGGAGEAGTATACAGCAAAC, *bgl*II-L 5′-3′: AATAGATCTAAGGACACTCGGATGATCTGTG. The PCR fragments of miR-195 were digested with *xho*I and *bgl*II. The inserts were ligated into the vector. Plasmid construction, molecular cloning, and packaging of lentiviruses were used. For lentiviruses silencing miR-195, the vector was pSico-RNAi-GFP. The sequences of the primers used for PCR were as follows: (5′-3′) shU: ACTGACTGACCAAACAGCAAAGTTCGACAGAGCACAGGACACAAGGCCTGTTAC, shL: GGCCAAAACCAAACAGCAATAGTTCGACAGAGCACAGCATACAGCCTCTAGCAA. The packaging procedure of lentivirus contains the following steps. 24 h before transfection, the 293T cells were seeded at a concentration of 1.5 × 10^6^ cells/mL in a 15 cm cell dish. Then, 2 h before transfection, the cell culture medium was changed to a serum-free medium. The DNA (pGC-LV vector 10 µg, pHelper 1.0 vector 10 µg, pHelper 2.0 vector 12 µg) was mixed with Opti-MEM and incubated for 5 min at room temperature. The Opti-MEM (2.4 mL) was then mixed with 100 µL of Lipofectamine 2000 and incubated for 3–5 min at room temperature. The diluted DNA and Lipofectamine 2000 were mixed and incubated and then added into the 293T cell medium. The transfection procedure of lentivirus contains the following steps: 18–24 h before transfection, the attached osteosarcoma cells were seeded at a concentration of 1 × 10^5^ cells/well in 24 well plates. The cell medium was changed into a new medium (2 mL) containing 6 mg/mL polybrene and then lentiviruses were added. After 4 h, 2 mL of medium was added to dilute the concentration of polybrene; 24 h later, the medium containing lentiviruses was changed.

### 2.7 MTT cell proliferation assay

We seeded cells at a concentration of 1 × 10^4^ cells/well with a total of 150 µL of the culture medium with FBS and antibiotics into 96-well microplates. Cell viability was checked after 1, 2, 3, 4, and 5 days. We conducted this assay by using the Cell Growth Determination Kit based on MTT (Sigma-Aldrich). In brief, we added 5 mg/mL MTT without phenol red in an amount equal to 10% of the culture volume (15 µL), incubated for 3 h, removed the culture fluid, dissolved the resulting MTT formazan crystals by adding 0.1 N HCl in isopropanol, mixed and spectrophotometrically measured the absorbance at a wavelength of 490 nm, and subtracted the background absorbance measured at 690 nm. All the wells were quadruple repetitions. Every four wells in 96-well microplates were manipulated in the same way. For each independent experiment, three 96-well microplates were used for detection on Day 1, Day 2, Day 3, Day 4, and Day 5 respectively.

### 2.8 Wound healing migration assay

Cells were cultured in 6-well tissue culture plates. A micropipette tip was used to scratch through the cell monolayer at the center of the plate. After two washes with phosphate-buffered saline, the medium was replaced with a medium containing 10% FBS. The cells were incubated for 24 h at 37°C and 5% CO_2_ and were imaged at 0 and 24 h.

### 2.9 Transwell invasion assay

Transwell invasion chambers (Corning Inc., Corning, NY) were coated with Matrigel (50 µL per filter) (BD Biosciences, Franklin Lakes, NJ). Cells (8 × 10^4^/200 µL/chamber) resuspended in a medium containing 10 g/L bovine serum albumin were added to the upper chamber, and 500 µL of the medium supplemented with 10% FBS was added to the lower chamber as a chemoattractant. After 24 h, the cells in the upper chamber were removed with a cotton swab, and those that had passed through the Matrigel-coated membrane were stained with 1% crystal violet (Solarbio, Beijing, China), photographed (200× magnification), and counted.

### 2.10 Statistical analysis

Statistical analysis was performed by an independent sample *t*-test, and *p* < 0.05 was considered statistically significant.

## 3 Results

### 3.1 Expression of miR-195 and FASN and the methylation level of miR-195 in the osteosarcoma tissues

**Figure 1:**
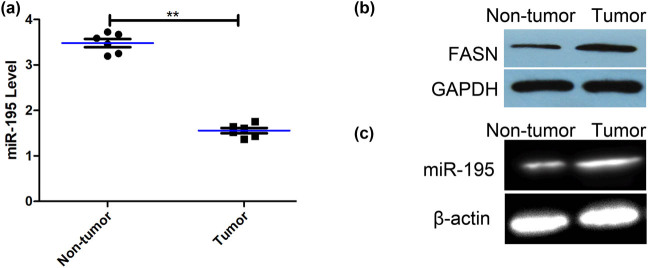
The expression of miR-195 and its methylation level in the osteosarcoma tissues. (a) The expression of miR-195 of fresh osteosarcoma tissues was significantly lower than that of fresh normal bone tissue (*n* = 6; *p* < 0.001). The error bar represents the standard variation of the miR-195 levels in patients with or without osteosarcoma. (b) The western blot assay showed that the expression of FASN of fresh osteosarcoma tissues was significantly higher than that of fresh normal bone tissues (*n* = 6). (c) MSP assay showed that the methylation levels of promoter regions of miR-195 in osteosarcoma tissues were significantly higher than that in fresh normal bone tissues (*n* = 6). *p < 0.05, **p < 0.01, *n* = 6/group.

The expression levels of miR-195 were significantly lower in the tissues of osteosarcoma than that in nontumors (Figure 1a). The protein expression of FASN of fresh osteosarcoma tissues was significantly higher than that of fresh normal bone tissue (Figure 1b). The methylation levels in the promoter region of miR-195 were significantly higher in the tissues of osteosarcoma than that in nontumors (Figure 1c).

### 3.2 miR-195 targeted and decreased the expression of FASN

**Figure 2:**
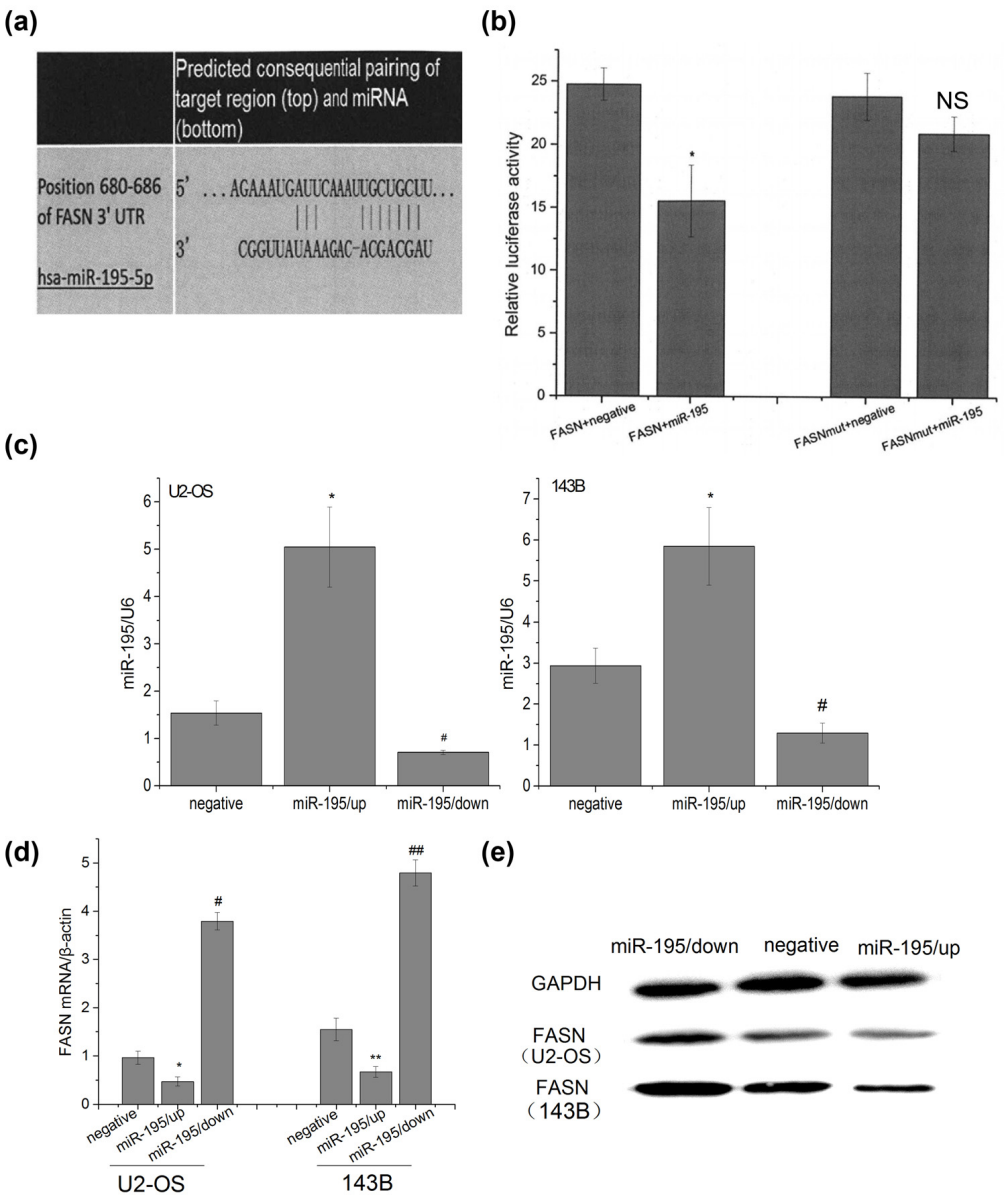
miR-195 targeted and decreased the expression of FASN. (a) Target Scan Human 6.0 software predicted that there was a target site of miR-195 on 680–686 bp of 3′-UTR of FASN. (b) Luciferase activity assay showed the following: the luciferase activity in the group psi-FASN Vector + miR-195 was significantly lower than that in the group FASN + negative controls (*n* = 6; *p* < 0.001), but there were no significant differences between the group psi-FASN mutVector + miR-195 and the group FASN mut + negative (*n* = 6: #*p* = 0.083), and the luciferase activity in the group psi-FASNmut Vector + miR-195 was significantly higher than that in the group psi-FASN Vector + miR-195 (*n* = 6; **p < 0.001). The error bar represents the standard variation of the luciferase activity in the cells transfected with FASN (or mutant FASN) plasmids and treated with or without miR-195. (c) The expression level of miR-195 was significantly up/downregulated in U2-OS and 143B cells transfected by miR-195/up or miR-195/down lentiviral vector (*n* = 6). The error bar represents the standard variation of the miR-195 levels in the U2-OS or 143B cells transfected with lentiviruses overexpressing or inhibiting miR-195. (d) The FASN mRNA expression of osteosarcoma cells decreased significantly in the group miR-195/up and significantly upregulated in the group miR-195/down *in vitro* by RT-PCR analysis (*n* = 6). The error bar represents the standard variation of the FASN levels in the U2-OS or 143B cells transfected with lentiviruses overexpressing or inhibiting miR-195. (e) The FASN protein expression of osteosarcoma cells decreased significantly in the group miR-195/up and significantly upregulated in the group miR-195/down *in vitro* by western blot analysis (*n* = 6).

The bioinformatics prediction tool Target Scan Human 6.0 indicated that there was a binding site between the miR-195 and 3′-UTR of FASN (Figure 2a). Luciferase assay verified that in the cells transfected with FASN 3′-UTR plasmids and miR-195, the luciferase activity decreased (Figure 2b), while the mutant plasmids had no effects, suggesting that miR-195 specifically targeted FASN. Next, we constructed lentiviruses silencing or overexpressing miR-195, and our transfection was successful (Figure 2c). miR-195 overexpression suppressed expression of FASN while miR-195 inhibition upregulated FASN at both mRNA and protein levels in both U2-OS and 143B cells (Figure 2d and e).

### 3.3 miR-195 inhibited cell proliferation, cell migration, and invasion in osteosarcoma

**Figure 3:**
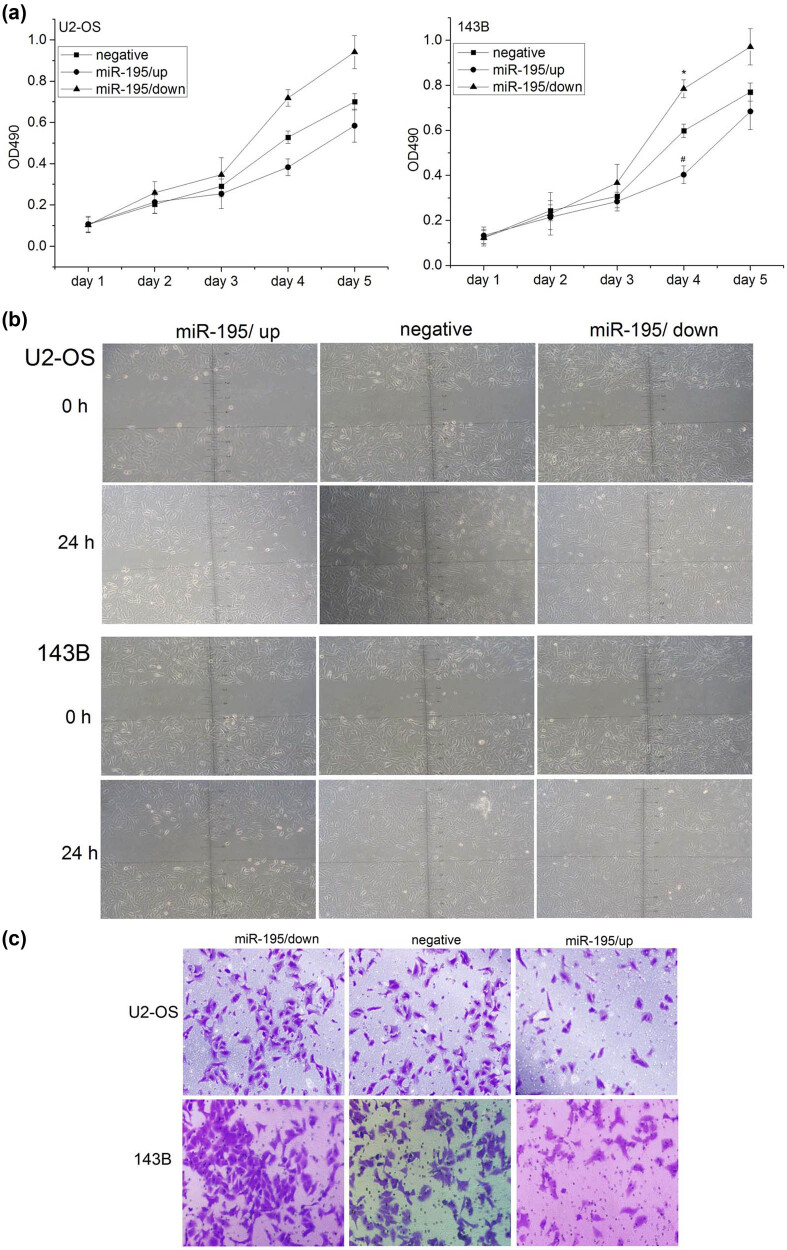
miR-195 inhibited cell proliferation, cell migration, and invasion in osteosarcoma. (a) The proliferation activity of osteosarcoma cells was detected by MTT assay. Left: the proliferation ability of U2-OS cells was significantly decreased in the group miR-195/up (*n* = 6; day 4: *p < 0.001) and significantly increased in the group miR-195/down *in vitro* (*n* = 6; day 4: #*p* < 0.001). Right: the proliferation ability of 143B cell was significantly decreased in the group miR-195/up (*n* = 6; day 4: *p < 0.001) and significantly increased in the group miR-195/down *in vitro* (*n* = 6; day 4: #*p* < 0.001). The error bar represents the standard variation of the OD490 in the U2-OS or 143B cells transfected with lentiviruses overexpressing or inhibiting miR-195. (b) The migration ability of osteosarcoma cells at 24 h using wound healing assay was significantly decreased in the group miR-195/up (*n* = 6, ×200). (c) The invasion ability of osteosarcoma cells at 24 h using Transwell invasion assay was significantly decreased in the miR-195/up group and significantly increased in the miR-195/down group (*n* = 6, ×200).

We then studied the functions of miR-195 in osteosarcoma cells. Silence of miR-195 promoted cell proliferation while overexpression of miR-195 inhibited cell proliferation in both U2-OS and 143B cells (Figure 3a). Overexpression of miR-195 also leads to reduced cell migration in U2-OS and 143B (Figure 3b). The reduced function of miR-195 increased cell invasion while the gain of function of miR-195 inhibited cell invasion (Figure 3c).

### 3.4 Methylation of miR-195 was one of the reasons why miR-195 decreased

**Figure 4:**
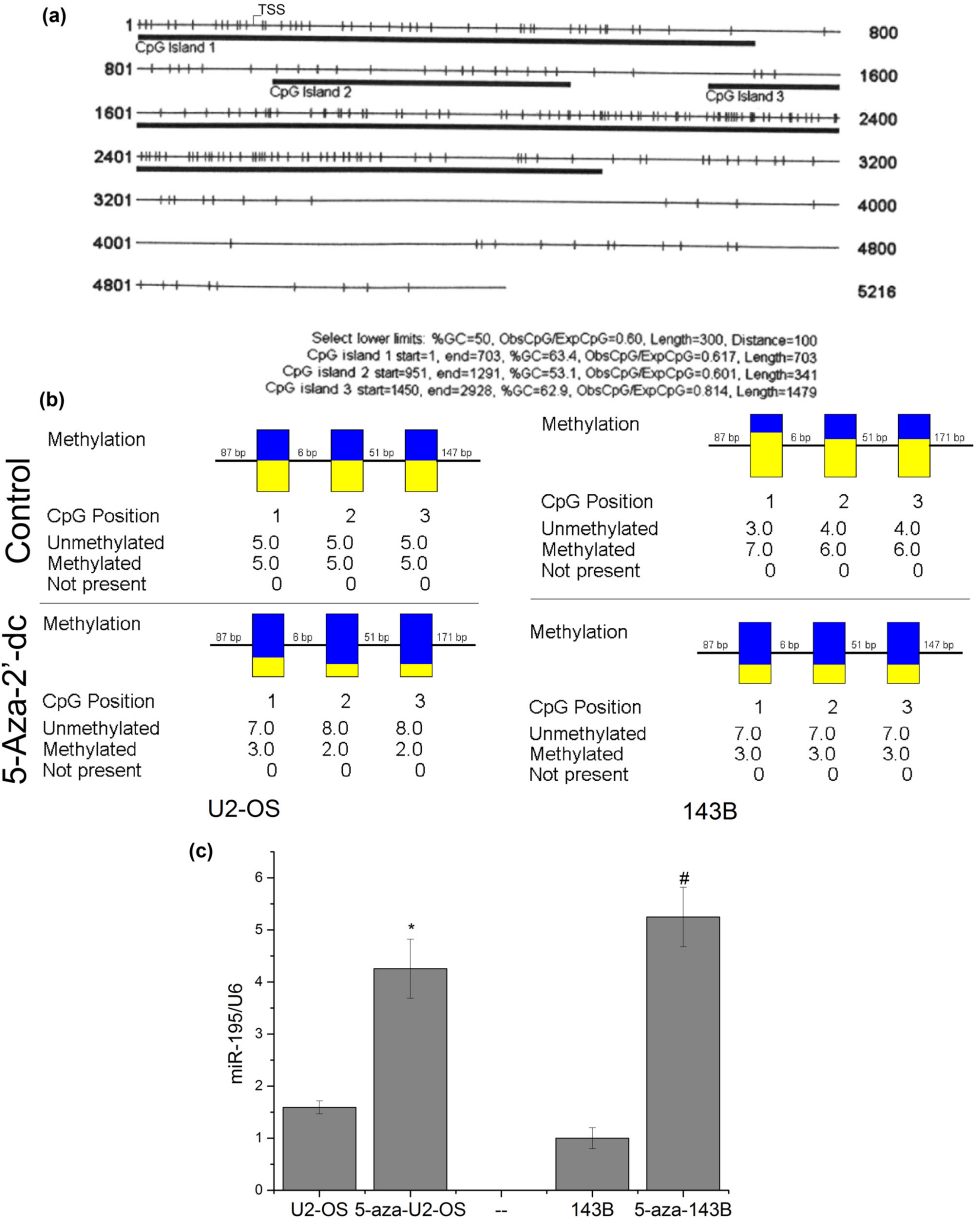
The methylation of miR-195 was one of the reasons why miR-195 decreased. (a) The prediction results showed that the promoter region of miR-195 is full of CPG islands. (b) Bisulfite sequencing PCR (BSP) assay showed that the methylation levels of the miR-195 promoter region were significantly downregulated on cells treated with 5-aza-2′-dc (*n* = 6). (c) The expression level of miR-195 was significantly upregulated in U2-OS and 143B cells treated with the demethylation reagent *in vitro* (*n* = 6). The error bar represents the standard variation of the miR-195 levels in the U2-OS or 143B cells treated with 5-aza.

We used the CpG island online prediction tool (http://cpgislands.usc.edu) to explore the possibility of methylation of the promoter region of miR-195 and found three CpG islands in the promoter of miR-195 (Figure 4a). We used methylation inhibition reagent 5-aza-2′-dc to treat U2-OS and 143B cells and detected the methylation level. The results showed that the methylation level significantly decreased after the drug was used (Figure 4b). The expression levels of miR-195 increased significantly after the cells were treated with 5-aza-2′-dc (Figure 4c), indicating that the methylation of the promoter region of miR-195 was one of the reasons why miR-195 decreased in osteosarcoma.

### 3.5 Methylation might promote osteosarcoma cell proliferation, migration, and invasion

**Figure 5:**
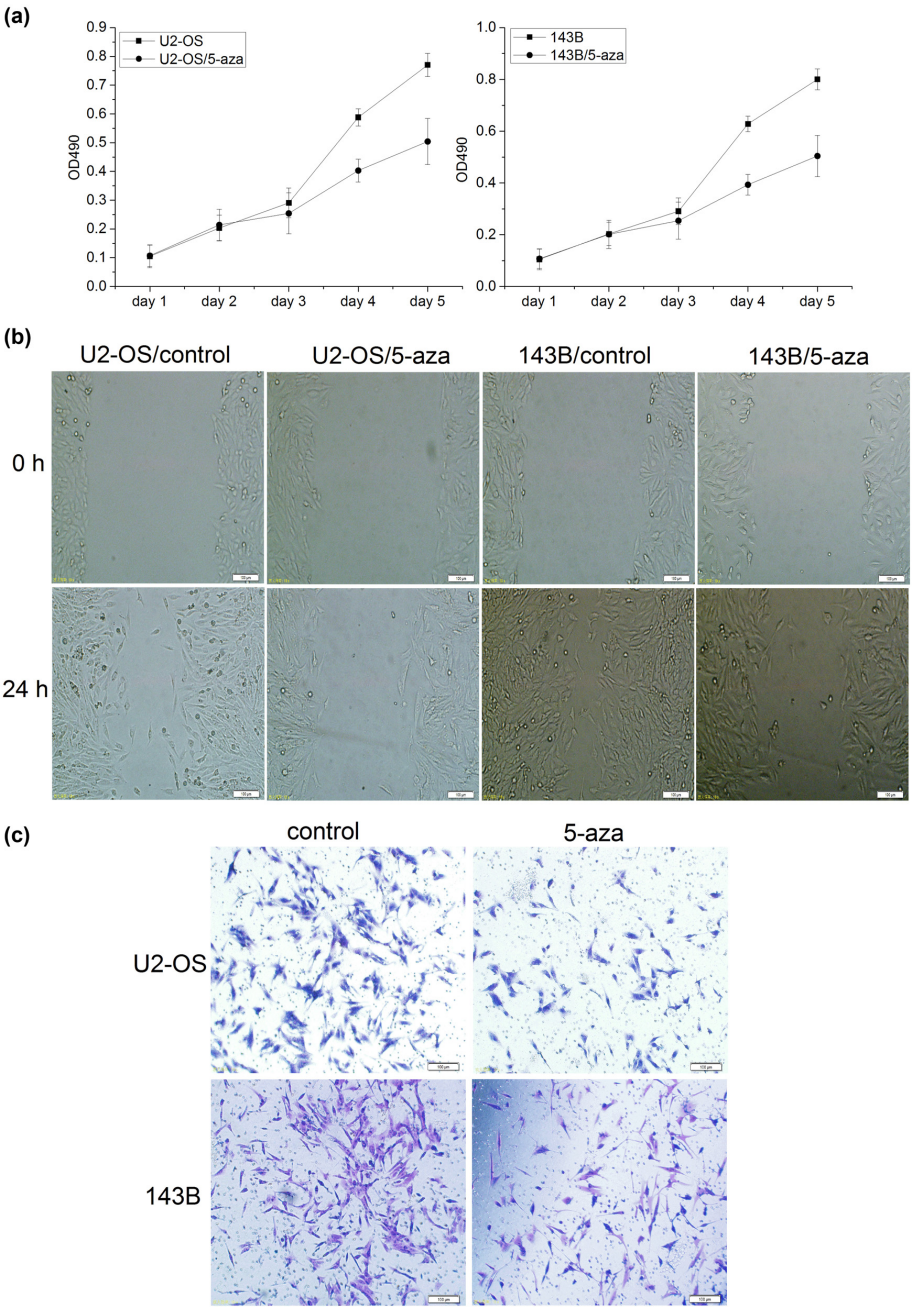
Methylation might promote osteosarcoma cell proliferation, migration, and invasion. (a) The proliferation activity of osteosarcoma cells was detected by MTT assay. MTT assay showed that the proliferation was significantly decreased in U2-OS and 143B cells treated with 5-aza-2′-dc (*n* = 6, day 4: *p* < 0.001). The error bar represents the standard variation of the OD490 in the U2-OS or 143B cells treated with 5-aza. (b) The migration ability of osteosarcoma cells at 24 h was significantly decreased in the cells treated with 5-aza-2′-dc (*n* = 6, ×200). (c) The invasion ability of osteosarcoma cells at 24 h was significantly decreased in the cells treated with 5-aza-2′-dc (*n* = 6, ×200).

MTT assay showed that cell proliferation decreased significantly after 5-aza-2′-dc treatment (Figure 5a). The migration ability of cancer cells at 24 h was significantly decreased in U2-OS and 143B cells treated with 5-aza-2′-dc (Figure 5b). Transwell invasion assay demonstrated the invasion ability of cells was significantly decreased in U2-OS and 143B cells after 5-aza-2′-dc treatment (Figure 5c). Taken together, these results suggested that the cell proliferation, migration, and invasion decreased after the cells were treated with the demethylation reagent.

## 4 Discussion

### 4.1 Summary

**Figure 6:**
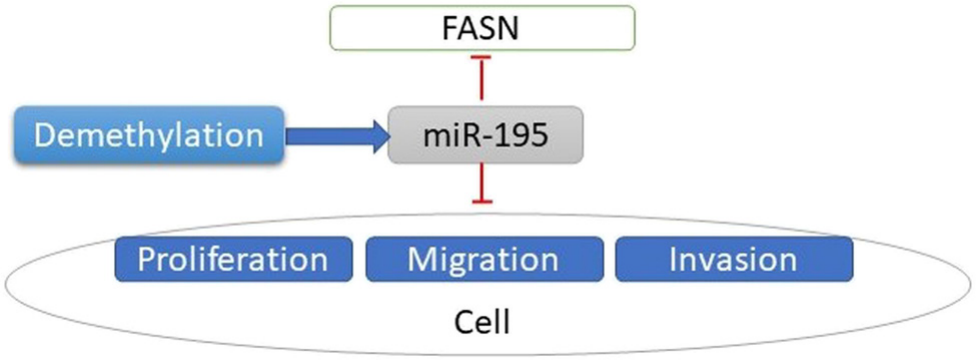
Flow chart of the summary of the main contents. Decreased expression of miR-195 mediated by hypermethylation promotes osteosarcoma.

Our previous studies showed FASN was an oncogene. In this study, we first found the expression of FASN was reversely correlated with the expression of miR-195. Then, we demonstrated that FASN was targeted by miR-195, which inhibited osteosarcoma cell proliferation, migration, and invasion. Finally, we used the demethylation reagent to treat the cells and miR-195 increased, indicating that in cancer the expression of miR-195 was suppressed by hypermethylation. The above studies are summarized in Figure 6.

### 4.2 Current therapy of osteosarcoma

Currently, the combination of surgery and multiple chemotherapies is the standardized treatment for osteosarcoma. However, there are low responses to drugs in many patients [26]. Chemotherapy is often administered before surgery to prevent tumors from spreading throughout the body. However, patients with distant metastases still fare poorly, as the 5 year survival rate is about 20% [27,28]. To date, no single genetic target has proven therapeutically successful [7]. Thus, attention should be paid to the underlying molecular mechanisms, which can serve as therapeutic targets. In this study, we identified FASN and miR-195 to be the potential therapeutic targets for osteosarcoma.

### 4.3 FASN

Nontumor cells naturally acquire palmitate from dietary and quickly store it in triglyceride pools. Palmitate together with other saturated fatty acids has lipotoxicity [29]. To avoid this issue, no-tumor cells change the saturated fatty acids to triglycerides, thereby storing the energy and deactivating the adverse effects [30]. In contrast, tumor cells vigorously synthesize fatty acids and store very little in triglycerides. Cancerous cells typically use these fatty acids to produce phospholipids to build membranes supporting cell proliferation [31].

A high level of fat in diets has been reported in the development of many malignancies [32,33]. Breast cancer cells exhibit high expression levels of FASN and activity [34]. The overexpression of FASN is significantly detected in many types of tumors, including lung, stomach, prostate [35], ovary, bladder, oral cavity, and melanoma [36]. In other words, FASN is expressed at high levels in a variety of human tumors.

Our previous studies show that the positive expression rate of FASN in patients with lung metastasis is 86.4% but only 52.2% in those without lung metastasis [37]. We also found that the expression of FASN was significantly correlated with the tumor size. The FASN levels were higher in tumor diameter larger than 8 cm than in smaller than 8 cm [37]. In this study, based on our previous study on FASN, we further study the miRNA regulating FASN. Specifically, the miR-195 could target and inhibit FASN.

### 4.4 Functions of FASN in tumors

Tumors have the ability to synthesize their own fatty acid, independent of the regulatory signaling pathways inhibiting the fatty acid synthesis in normal cells [8]. All esterified fatty acids in the tumor are from de novo synthesis [38]. Endogenous fatty acid synthesis is a significant source of fatty acids for the growth of tumor cells [8]. The synthesis of fatty acids is essential for the biogenesis of cellular membranes in rapidly dividing cancer cells [39]. Long-chain fatty acids generated from the palmitate precursors (in turn generated by FASN) are essential for cell division [39,40]. Inhibition of FASN decreased the DNA synthesis and suppressed cells to progress to S-phase [41]. De novo synthesis of membrane phospholipids might be necessary for cell dividing.

The tumors with high expression levels of FASN have increased palmitoylation of Wnt-1 and stabilized β-catenin [29], which might activate the transcription factor. Besides Wnt, tubulin and Harvey rat sarcoma viral oncogene homolog (HRAS) require palmitoylation to localize and function appropriately [42]. In addition, the PI3K/Akt/mTOR pathway and protein kinase C depend on FASN [43]. It is reported that inhibition of FASN results in a decline in diacyl glycerols, those in which at least one acyl chain is palmitate. The decrease of diacyl glycerols leads to a decrease in the protein kinase C pathway, resulting in the death of the tumor cell [44]. Flow cytometry has demonstrated that, due to the inhibition of fatty acid synthesis, cells accumulated in G1, indicating a relation between FASN and cell cycle [45]. Inhibition of FASN leads to an inhibition of S-phase progression and DNA replication [41].

### 4.5 FASN and metastasis of osteosarcoma

During the synthesis of endogenous fatty acids, the key enzyme, FASN, is responsible for catalyzing the synthesis of long-chain fatty acids in mammals. Also, FASN is critical in sustaining the biological features of malignant tumor cells [46]. In fact, FASN has been studied as a candidate oncogene in cancer [47] such as prostate cancer [48], liver cancer [49], and ovarian cancer [50]. Recently, evidence has shown that fatty acid metabolic pathways play a critical role in carcinogenesis [51]. Inhibition of FASN expression could suppress malignant tumor cell proliferation *in vitro* and *in vivo* in oral squamous cell carcinomas [52], liver cancer [53], and neurogenesis [54]. Therefore, FASN has been considered as a promising target for anticancer treatment and management. However, the molecular roles of FASN in osteosarcoma cells remain unclear and need to be further studied.

Increasing evidence showed that FASN also contributes to colorectal cancer cell metastasis [55]. However, whether FASN could promote metastasis in OS and the molecular experimental mechanisms remain unclear. One of the most important reasons for lung metastasis is anoikis resistance [56]. Whether FASN assists lung metastasis of OS by enhancing the anoikis resistance and the detailed molecular and cellular mechanisms need to be elucidated.
